# Long-term organic fertilizer additions elevate soil extracellular enzyme activities and tobacco quality in a tobacco-maize rotation

**DOI:** 10.3389/fpls.2022.973639

**Published:** 2022-09-09

**Authors:** Yonglei Jiang, Ruqiang Zhang, Cuiping Zhang, Jiaen Su, Wen-Feng Cong, Xiaopeng Deng

**Affiliations:** ^1^Yunnan Academy of Tobacco Agricultural Sciences, Kunming, China; ^2^Key Laboratory of Plant–Soil Interactions, Ministry of Education, College of Resources and Environmental Sciences, National Observation and Research Station of Agriculture Green Development (Quzhou, Hebei), China Agricultural University, Beijing, China; ^3^Yuxi Branch, Yunnan Tobacco Co., Ltd., Yuxi, China

**Keywords:** organic fertilizer, soil enzyme activity, crop quality and yield, rotation cropping, tobacco

## Abstract

Organic fertilizer is effective in improving soil quality, and promoting crop growth. Combined organic and inorganic fertilization has been proved as a more favorable way to tobacco yield and quality. However, the mechanisms underlying tobacco yield and quality under combinations of different organic and inorganic fertilizer remain unclear. We conducted a 12-year tobacco (*Nicotiana tabacum* L.)-maize crop rotation field experiment in Yanhe experimental station, China to examine the yields and qualities of tobacco, soil nutrients, and extracellular enzyme activities associated with carbon, nitrogen, and phosphorus cycles in response to different fertilization treatments. Five fertilization treatments (no fertilization; 75 kg N fertilizer ha^–1^; 450 kg oil cake ha^–1^ + 75 kg N fertilizer ha^–1^; 15,000 kg pig dung ha^–1^ + 60 kg N fertilizer ha^–1^; 3,000 kg straw ha^–1^ + 75 kg N fertilizer ha^–1^) were applied to tobacco while maize was fertilized with inorganic compound fertilizers. After 12 years of tobacco-maize rotation, the results showed that organic fertilizer additions elevate tobacco yield and quality, and the soil extracellular enzymes activities. Gram-negative bacteria, actinomycetes, and total soil microbial biomass were increased by organic fertilizer additions, both plant-based (oil cake and straw) and animal-based (pig dung) organics. The levels of soil organic matter, total organic carbon, total phosphorus and available phosphorus are higher in pig dung addition treatment than oil cake and straw additions. By variance analysis with respect to fertilization treatments, organic sources differentially affected the activities of diverse soil enzymes. The redundancy analysis gave that yield and quality of tobacco leaves (upper, middle, and lower leaves) positively related to soil extracellular enzyme activities. Based on analysis of yield and quality of tobacco leaves with extracellular enzyme activities and soil nutrients, it is suggested animal-based organic fertilizer, thus pig dung, should be used in combining with chemical fertilizers to improve the quality of tobacco and soil nutrients.

## Introduction

Tobacco (*Nicotiana tabacum* L.) is an internationally distributed economic crop, where countries such as China, United States, Brazil, India, and Turkey, etc., are the most important planting and production areas (Drope et al., 2018). The yield and quality of tobacco is influenced by various factors, such as fertilization and crop rotation patterns, effecting plant growth and development and improving the chemical and physical characteristics of flue-cured leaves (Zou et al., 2015; Jiang et al., 2022). The optimized fertilization, and rotation with maize are effective in alleviating consecutive monoculture cropping problems while not affects the production in the coming year (Turšić et al., 2005; Farooq et al., 2014; Chen et al., 2020). In the fertilizer strategies, the partially organic substitution, or combined organic and inorganic fertilization, is more favorable in improving soil quality and increasing crop yield (Liu et al., 2022).

The combined application of inorganic fertilizers with organic materials is effective to crop yields and sustainable agroecosystems (Berzsenyi et al., 2000; Saleque et al., 2004). Organic resources, including animal manures from pigs, cows, chickens, or various others, household composts like meals of bone, feather, or fish, etc., crop residues like straw, and meal of maize gluten, soybean, or cottonseed, leguminous cover crops, trees and shrubs, are major nutrient sources to crops. The oil cake from residue of rape (*Brassica napus)* seed after extracting oil is widely applicated in tobacco planting. The differences in organic sources may result in differential yield responses to organic resource (Palm et al., 2001). How different sources of organic fertilizers combing with chemical fertilizers, and the proportion of their combination affect the agronomic traits, yield and quality of crops or soil fertility, is essential to take advantages of various organic fertilizers (Chivenge et al., 2011; Zhang et al., 2016; Lourenço et al., 2020).

The effects of combined organic and inorganic fertilizers on soil quality are critical to crop yield and quality (Liu et al., 2022). The overzealous pursuit of high tobacco yields and the heavy use of chemical fertilizers is not only detrimental to the plant’s intrinsic nutritional qualities, but it also wastes a lot of chemical fertilizer resources. The use of combined organic and inorganic fertilizers can help to alleviate these issues by improving the soil quality, such as soil organic matter, bulk density, porosity, soil buffer capacity and other soil traits, as well as improving soil microbe activity (Tian et al., 2015). The global need for nitrogen fertilizer in agriculture for crop production are increasing (Galloway et al., 2008), inducing to decline of N use efficiency, and impairing the soil microbes below ground (Su et al., 2015). These indicate the implications and impendency to develop the organic fertilizer additions.

To reveal the mechanism of organic fertilizer additions to crop yield and quality *via* changing soil quality, the soil processes, such as the nutrient cycling and organic matter decomposition, etc., are involved, which can be indicated by the rhizosphere microbial biomass and extracellular enzyme activity (Zheng et al., 2020). Soil extracellular enzymes, derived from soil microbes, roots, and animal and plant residues, can indicate microorganism activity and characterize soil fertility (Bell et al., 2013). The impact of fertilization on extracellular enzyme activity is influenced by a variety of factors, including fertilizer used, the crop planted, and the soil type. Extracellular enzymes are increased by organic fertilizer because it relieves nutrient restrictions for microbes (Lupwayi et al., 2019). Hydrolases related to C and P acquisition, while not to N acquisition, can be increased by N addition, according to a meta-analysis based on 65 published N fertilization trials (Jian et al., 2016). In a wheat-maize cropping system, the activities of acid phosphatase (AP), L-leucine aminopeptidase (LAP), and β-1, 4-N-acetyl-glucosaminidase (NAG) can significantly be altered by long-term application of inorganic fertilizers and manure (Sehrish et al., 2021). Therefore, we investigated how varied fertilization affects the rhizosphere soil microbial community and extracellular enzyme activities in soils.

In the tobacco-maize rotation, which of widespread cultivation system in Yunnan province, however, little effort has been expended on how soil extracellular enzyme activity respond to long-term organic fertilizer additions in tobacco-maize rotation. The long-term effects of management practices on soil and crops, such as the content of soil organic matter (SOM) were investigated after various years of management inputs (Liu et al., 2021). Therefore, we investigated the tobacco yield and quality, rhizosphere soil extracellular enzyme activities, and soil nutrients in tobacco-maize rotation soils that had been fertilized with organic (oil cake, pig manure, and straw) and inorganic fertilizers for 12 years. We hypothesized that (1) long-term application of inorganic fertilizer would decrease the content of SOM, and increase the accumulation of P and K elements in soil; (2) the combined application of inorganic and organic fertilizers would increase the SOM content and the activity of extracellular enzymes involved in C, N, and P cycling; and (3) animal organic fertilizer would more conducive to the improvement of soil organic matter and phosphorus compared with plant organic fertilizer. In order to distinguish the organic sources, the efficacy of plant-based and animal-based organic fertilizers was compared in aspects of yield and quality of tobacco, extracellular enzyme activities, and soil nutrient contents. From the perspectives of soil extracellular enzymes, we have investigated the mechanisms of fertilizations to tobacco’s yield and quality, the organic matter, carbon, nitrogen, and phosphorus of rotational soils, and to provide some theoretical basis for nutrient management practices in the tobacco cropping system.

## Materials and methods

### Site characteristics

The experimental site was located at the Yanhe experimental station in Yanhe Town, Yuxi City, Yunnan Province (N 24°14′, E 102°30′), with an altitude of 1680 m and annual average sunshine of 2072 h, in the plateau subtropical monsoon climate region. The soil in study area was a sandy red soil (silty loam), which is composed of 28% sand, 50% loam, and 22% clay. This soil type is an Ultisol in Soil Survey Staff (2014) soil taxonomy. Initial soil fertility of the experiment was a pH of 6.4, and had 10.70 g Kg^–1^ organic matter, 82.0 mg kg^–1^ available nitrogen (AN), 70–90 mg kg^–1^ alkali-hydrolyzable N, 9.01 mg kg^–1^ available phosphorus (AVP), and 160.0 mg kg^–1^ available potassium (AK). The annual average rainfall is 800–950 mm. The variation in mean monthly precipitation is wide and the rainy season (from April to September) accounts for 79.5% of the annual rainfall. The average annual temperature is 15.9^°^C. The average monthly temperature difference between the hottest and coldest months is generally less than 10^°^C.

### Field experiments and sample collection

The rotated tobacco variety was *Nicotiana tabacum* L. “K326.” As shown in Table 1, the fertilizer management in this experiment was divided into inorganic nitrogen fertilizer and three organic fertilizers applied in combination with oil cake, pig manure after composting and fermenting and straw (see Supplementary Table 1 for nutrient contents of organic additions), with five treatments and three replications per treatment in a randomized block design with a plot size of 14 m and a width of 2 m. From 2008 to 2019, the tobacco-maize crop was rotated every 2 years, where tobacco and maize were planted in each summer (from March to July), and land were free in winter. The doses of combined organic and inorganic fertilizers to tobacco were established in 2007 (Chen et al., 2020). The inorganic fertilizer to maize was a compound fertilizer with nitrogen of 16 kg (35 kg of urea, 46% of nitrogen), P_2_O_5_ of 5.4 kg (30 kg calcium superphosphate, 18% of phosphorus) and K_2_O of 5 kg (10 kg potassium sulfate, 50% of K_2_O) and applied as 75 kg ha^–1^ in total in one season. In these experiments, 50% of the fertilizer was used as a basal fertilizer strip around the tobacco plants. Approximately 25% of the fertilizer was applied at 7 and 20 days after tobacco transplanting, respectively. Biomass residues, including roots, were completely removed after tobacco harvesting. Other agronomic measures were implemented according to the quality roasted tobacco production measures developed by the Comprehensive Technology Extension Center of Yunnan Academy of Tobacco Agricultural Sciences.

**TABLE 1:** Experimental design.

Treatment	Organic fertilizer	Inorganic nitrogenous fertilizer
Oil cake (OC) (plant)	Oil cake 450 kg/ha	75 kg N/ha
Pig dung (PD) (animal)	Pig dung 15000 kg/ha	60 kg N/ha
Straw (S) (plant)	Straw 3000 kg/ha	75 kg N/ha
Compound fertilizer (CF) (inorganic fertilizer)	None	75 kg N/ha
Control (CK)	None	None

The soil samples were collected at August 2019, and five soil samples, each from 0 to 20 cm were randomly taken and mixed in each plot using the profile method. After bringing them back to the laboratory to remove debris such as roots and small stones from the soil, they were placed in self-sealing bags and set aside in a refrigerator at –80°C.

All tobacco leaves were collected at July, 2019 and the dry matter content was determined to calculate the yield after tobacco leaves were first flue-cured. The leaves samples were collected after their maturity. The tobacco leaves of a plant, generally left 18–22 leaves after topping, are divided into three parts: upper, middle, and lower. The upper leaves include the upper two shed leaves and the top leaves, totaling 6–7 leaves; the middle leaves, the waist leaves, totaling 6–8 leaves; and the lower leaves, the lower two shed leaves and the foot leaves, totaling 6–7 leaves. The maturity and harvesting time of different parts of the tobacco leaf varies, with the lower leaves reaching maturity generally 60–70 days after transplanting and 10–20 days after topping; the middle leaves generally 80–90 days after transplanting and 20–30 days after topping; and the upper leaves 90–120 days after transplanting and 45–65 days after topping.

### Chemical analysis

To determine the nutrient contents in first flue-cured tobacco leaves, 1 kg leave sample was randomly taken in each treatment, in which the main veins were removed, and grinded and sieved through a 0.4 mm sieve before analyzing the chemical components (Sun et al., 2011). The alkaloids in tobacco leaves including nicotine, nornicotine, myosmine, *N*-methyl anabasine, β-nicotyrine, anabasine, anatabine, isonicotenine and cotinine, were determined by gas chromatography-mass spectrometry method (Li et al., 2019) and their contents were summed to calculate ratio of sugar to alkaloids.

Soil organic C and total N were determined by the potassium dichromate volumetric method (Kalembasa and Jenkinson, 1973) and semimicro-kjeldahl method (Bremner and Mulvaney, 1982), respectively. The soil inorganic nitrogen (${\text{NH}}_{4}^{+}$ and NO${}_{3}^{-}$) contents were measured by copperized cadmium reduction-diazotization coupling colorimetric method. Total P was determined by the NaOH fusion-molybdenum antimony colorimetric method and available P was analyzed by anion-exchange resin method, respectively. Soil exchangeable cations were determined by ammonium acetate exchange method.

### Extracellular enzyme activities determination

Six potential soil extracellular enzymes, BG (β-1, 4-glucosidase), NAG (β-1, 4-N-acetyl-glucosaminidase), LAP (L-leucine aminopeptidase), AP (acid phosphatase), PER (peroxidase), and PPO (polyphenol oxidase) were determined by 96-well micro-plate fluorescence method (Vepsäläinen et al., 2001). The list of enzymes that were assayed and their corresponding substrates are presented in Supplementary Table 2. The extracellular enzyme activities were determined by a 96-microplate fluorometric method, and polyphenol oxidase and peroxidase were determined by red purpurine colorimetry and pyrogallol colorimetry, respectively (Vepsäläinen et al., 2001).

Glomalin-related soil protein (GRSP) determination: T-GRSP (total glomalin-related soil protein) and EE-GRSP (easily extracted glomalin-related soil protein) were extracted with sodium citrate solution and determined by Komas Brilliant Blue method (Serra and Morgante, 1980).

### Microbial biomass and community determination

Microbial biomass carbon, nitrogen, and phosphorus were determined by the chloroform fumigation extraction method (Vance et al., 1987). Soil microbial community composition was determined by the phospholipid fatty acid (PLFA) biomarker method, referring to the method of Bossio (Frostegård et al., 1991; Bossio and Scow, 1998), which was partially determined at the South China Institute of Botany, Chinese Academy of Sciences. Fatty acids 14:00, 16:00, 17:00, and 18:00 were considered as general bacteria, while Gram-positive bacteria included fatty acids i13:0, i14:0, i15:0, a15:0, i16:0, a17:0, and i17:0; Gram-negative bacteria include fatty acids 14:1ω5c, 16:1ω7c, 17:1ω8c, 17:1ω9c, 18:1ω5c, 18:1ω7c, 18:1ω9c, cy17:0, and cy19:0 (Hortal et al., 2013). The fatty acids 10Me17:0 and 10Me18:0 was labeled as actinomycetes (Waldrop et al., 2004; Schmitt and Glaser, 2011). Fatty acids 16:1ω11c and 18:2ω6c were labeled as fungi (Hortal et al., 2013), and fatty acids 20:0 and 20:4ω6,9,12,15c were labeled as protozoa (Mauclaire et al., 2003).

### Data statistics and analysis

For each variable that was measured, including tobacco’s yield and quality, various extracellular enzyme activities, SOM, C, N, and P, and microbial biomass in PLFAs, the data were analyzed by one-way analysis of variance (ANOVA) using Fisher’s least significant difference (LSD) at *P* ≤ 0.05. Redundancy analysis (RDA, by “vegan” package), and data visualization (by “ggplot2” package) were carried out in R v4.2.0 version.

## Results and discussion

### The yields and qualities of tobacco

**FIGURE 1:**
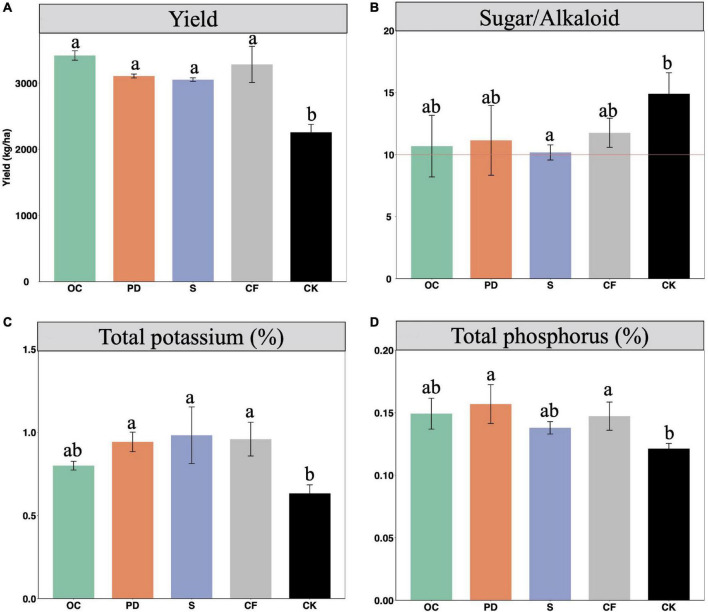
The yields **(A)** and qualities, including ratio of sugar to alkaloids **(B)**, total potassium **(C)**, and total phosphorus **(D)** of tobacco under different fertilization management after 12-year tobacco-maize rotation, where the combined addition of fertilization includes oil cake and inorganic nitrogen (OC), pig dung and inorganic nitrogen (PD), straw and inorganic nitrogen (S), inorganic nitrogen (CF), and no fertilization (CK). The yield is the dry matter weight of leaves after first flue-cured. Tobacco with the ratio value of sugar over alkaloid 10 is considered as the most proper. Data are means ± SE. Significant differences were determined by ANOVA, Fisher’s least significant difference (LSD) test (*P* < 0.05) and indicated by different letters.

The application of fertilizers significantly increased the yields of tobacco (Figure 1A). The highest yields of tobacco are in OC addition, while the differences between the yields of four different fertilizations are not significant. CF fertilizer could improve yield more effectively in comparison with PD addition and S addition.

The quality of tobacco are determined by its nutrient levels in tobacco leaves (Stedman, 1968). The contents or values, including the ratio of sugar over alkaloid, the total potassium, and total phosphorus, etc., are used to indicate the qualities of tobacco, which are shown in Figures 1B–D. The ratio of sugar and alkaloids, value 10 is considered as a highest quality of tobacco. When no fertilization, this ratio value is about 14.90, while the fertilizations result in the value to be closely to value 10 (Figure 1B). In addition, fertilizers benefit the accumulation of the total potassium and phosphorus content in tobacco leaves.

The sensor quality, including aroma quality, aroma quantity, strength and smoke density, are negatively related to the ratio of sugar to alkaloids (Guo et al., 2014). When organic fertilizers are added, the decreased values of ratio of sugar to alkaloids indicate the improved sensor quality. The smoking gas indices of flue-cured tobacco are negatively influenced by potassium content, as well as the hazardous constituents in flue gas (Yang et al., 2011). The organic fertilizer additions indicate the smaller smoking gas indices and decreased hazardous constituents in flue gas. The yield and quality of flue-cured tobacco leaves improved by phosphorus, is *via* enhancing photosynthetic characteristics and tobacco growth (Cui et al., 2016). The increased phosphorus contents in organic fertilizer additions indicate that organic fertilizers facilitate tobacco yield through promoting photosynthetic.

The main and interactive effects in tobacco leaves quality as influenced by fertilization and position were shown in Supplementary Table 3. Organic fertilization significantly increased the levels of total sugar, alkaline, nitrogen, potassium, and phosphorus. The levels of total alkaloids and total nitrogen are significantly distributed in different parts of the tobacco plant. No significant differences in element levels resulted from the interactive effects of fertilization and parts.

### Soil organic matter, carbon, nitrogen, and phosphorus

After 12-year tobacco-maize rotation, the highest SOM, total organic carbon (TOC), total nitrogen (TN), total phosphorus (TP), available phosphorus (AVP), available potassium (SK), and cation exchange capacity (CEC) were found in PD addition, and the content of SOM, TOC, TP, and AVP in PD addition were significantly greater than in the other three fertilizer addition and no fertilizer (*P* < 0.05) (Supplementary Figure 2 and Table 2). The content of ${\text{NH}}_{4}^{+}$-N and electric conductivity (EC) in CF application was the maximum and markedly greater than in S addition and OC addition, respectively. However, no significant difference in TK and NO${}_{3}^{-}$-N contents for all fertilization, among which CF application leads to the highest TK, while the highest NO${}_{3}^{-}$-N in no fertilization. The soil microbial biomass carbon, nitrogen and phosphorus are list in Supplementary Table 5, after 12-year tobacco-maize rotation, where no significant difference were detected in these variables.

**TABLE 2:** Effects of different fertilization management on soil organic matter, soil and microbial carbon, nitrogen and phosphorus after 12-year tobacco-maize rotation.

Fertilization management	SOM (g/kg^–1^)	TOC (g/kg^–1^)	TN (g/kg^–1^)	TP (g/kg^–1^)	${\text{NH}}_{4}^{+}$ (g/kg^–1^)	AVP (g/kg^–1^)	EC (%)	MBC:P	MBN:P
OC	14.1 ± 0.4B	8.2 ± 0.2B	0.9 ± 0.06B	1.3 ± 0.08B	2.1 ± 0.5AB	58.4 ± 1.2B	145.5 ± 18.4B	43.7 ± 7.2AB	19.2 ± 4.6AB
PD	16.0 ± 0.4A	9.3 ± 0.2A	1.2 ± 0.1A	1.5 ± 0.02A	2.9 ± 1.2AB	73.2 ± 1.9A	318.2 ± 66.3AB	33.8 ± 3.3Ab	12.1 ± 2.2B
S	13.5 ± 0.9B	7.8 ± 0.5B	1.2 ± 0.04A	1.1 ± 0.03B	0.3 ± 0.08B	47.7 ± 2.2C	225.5 ± 38.8AB	64.2 ± 6.5A	31.5 ± 4.8A
CF	13.0 ± 0.4B	7.5 ± 0.2B	1.0 ± 0.05AB	1.2 ± 0.03B	4.3 ± 1.1A	47.5 ± 0.5C	455.7 ± 88.2A	44.7 ± 19.6AB	16.6 ± 8.4AB
CK	13.6 ± 0.5B	7.9 ± 0.3B	1.1 ± 0.1AB	1.1 ± 0.1B	2.9 ± 1.2AB	40.6 ± 3.7C	339.4 ± 146.9AB	25.5 ± 8.7B	7.8 ± 2.6B

Value are means ± SE, n = 3. Different capital letters indicate significant among fertilizer treatments (P < 0.05). OC, oil cake and inorganic nitrogen; PD, pig dung and inorganic nitrogen; S, straw and inorganic nitrogen; CF, inorganic nitrogen; CK, no fertilization.

### Extracellular enzyme activity

**FIGURE 2:**
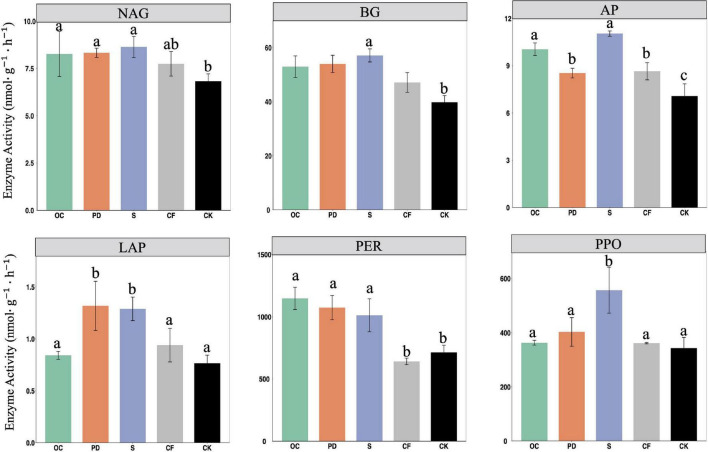
The activities of extracellular enzymes NAG, AP, BG, LAP, PER, PPO in soil under fertilizations oil cake (OC), pig manure (PD), straw (S), chemical (CF) and no fertilization (CK) after 12-year tobacco-maize rotation. See Supplementary Table 2 for abbreviations. Data are means ± SE. Significant differences were determined by ANOVA, Fisher’s least significant difference (LSD) test (*P* < 0.05) and indicated by different letters.

The activities of six measured extracellular enzymes in soil after 12-year tobacco-maize rotation under organic fertilizer additions of oil cake, pig manure, straw, chemical and no fertilization were shown in Figure 2. Organic fertilizers result in significantly greater NAG, AP, BG, and PER activities in soils in comparison with no fertilizer (*P* < 0.05). The increased activities of these six enzymes by S addition to no fertilizer are notably greater.

Organic fertilizer additions have significantly promoted the content of Total glomalin-related soil proteins (T-GRSP) after 12-year tobacco-maize rotation (*P* < 0.05), and its level reaches the highest value in the S addition (Supplementary Figure 1). The differences between the levels of easily extractable glomalin-related soil protein (EE-GRSP) in different treatments are not significant. It reached a maximum value in S addition while a minimum value in no fertilization (not shown).

The activities of BG, NAG, LAP, and AP were elevated by organic fertilizer additions (Figure 2), possibly as a result of elevated organic matter in soil and greater microbial activity and more enzymes (Zheng et al., 2020). The PER activity in soil was increased significantly by organic fertilizer addition. Organic fertilizer contains lignin and humus and is superior in enhancing PER activity since it degrades lignin (Sinsabaugh et al., 2009). The accumulation of SOM which benefiting from PPO catalyzing the oxidation of phenols, together with the inhibitory effect of inorganic and manure on PPO (Zhang and Sun, 2021), leads to the highest PPO activity in the S addition.

### Yield and quality of tobacco and soil extracellular enzyme activity

**FIGURE 3:**
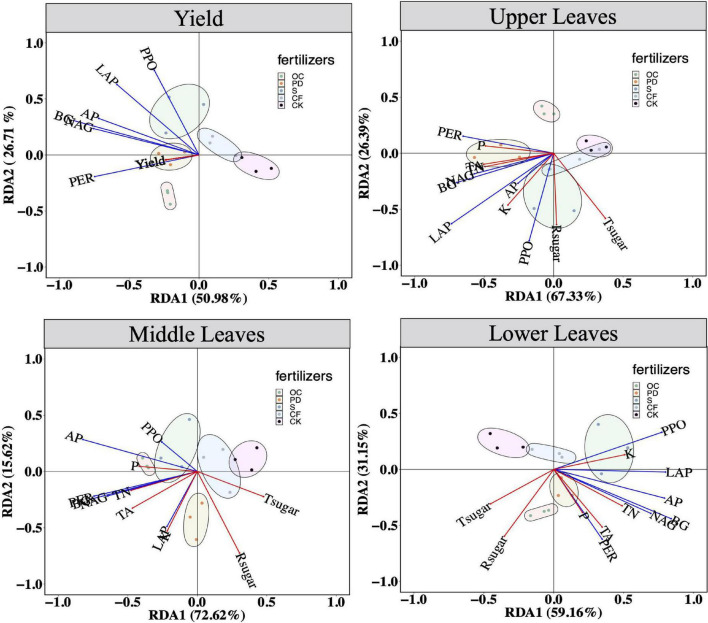
Redundancy analysis (RDA) of extracellular enzyme activity on yields, and the element levels in the up part, the middle part and lower part of tobacco, respectively, after 12-year tobacco-maize rotation. Tsugar, total sugar in tobacco of corresponding parts; Rsugar, reductive sugar; TA, total alkaloid; TN, total nitrogen; AP, available phosphorus; P, phosphorus; K, potassium; BG, β-1, 4-glucosidase; NAG, β-1,4-N-acetyl-glucosaminidase; LAP, L-leucine aminopeptidase; AP, acid phosphatase; PER, peroxidase; PPO, polyphenol oxidase. OC, oil cake and inorganic nitrogen; PD, pig dung and inorganic nitrogen; S, straw and inorganic nitrogen; CF, inorganic nitrogen; CK, no fertilization.

The relationships between the extracellular enzyme activity and the yield and qualities of tobacco leaves by RDA are shown in Figure 3. The first two axes of the RDA, RDA1, and RDA2, with 50.98 and 26.71%, explain rates of the variation in the relationship between yield and the extracellular enzyme activity. Organic fertilizer additions leads the tobacco yields are significantly differed from inorganic fertilizers and no fertilizers. The values of RDA1 and RDA2, or explain rates of the variation, are 67.33 and 26.39%, in the relationship between the qualities in upper leaves of tobacco and the extracellular enzyme activity, respectively. For the qualities in the middle leaves of tobacco and the extracellular enzyme activity, the explained rates of the variation are 72.62 and 15.62%, respectively. Those two values are 59.16 and 31.15% in the relationship between the qualities in the lower leaves of tobacco and the extracellular enzyme activity, respectively. In upper leaves, middle leaves, and lower leaves, the confidence ellipses of organic fertilizer addition are significantly separated from those in no fertilization. Briefly, yield and quality of tobacco leaves in upper, middle and lower positions, are all positively related to soil extracellular enzyme activity.

### Soil organic matter, carbon, nitrogen, phosphorus and soil extracellular enzyme activity

**FIGURE 4:**
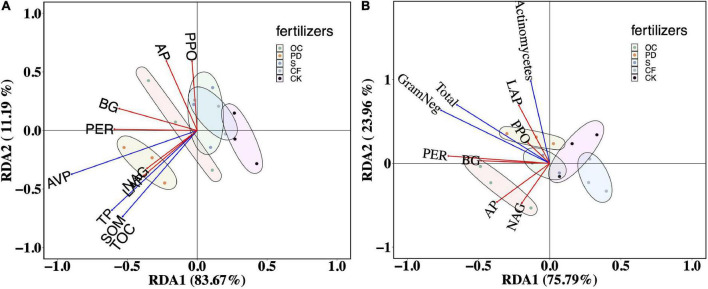
Redundancy analysis (RDA) of soil organic matter, carbon, phosphorus **(A)** and the contents of PLFAs **(B)** on extracellular enzyme activity after 12-year tobacco-maize rotation. SOM, soil organic matter; TOC, total organic carbon; TP, total phosphorus; AVP, available phosphorus; total organisms (Total), total Gram-negative bacteria (GramNeg), total *Actinomycetes* (Actinomycetes); BG, β-1, 4-glucosidase; NAG, β-1,4-N-acetyl-glucosaminidase; LAP, L-leucine aminopeptidase; AP, acid phosphatase; PER, peroxidase; PPO, polyphenol oxidase. OC, oil cake and inorganic nitrogen; PD, pig dung and inorganic nitrogen; S, straw and inorganic nitrogen; CF, inorganic nitrogen; CK, no fertilization.

How biochemicals in the soil to constrain the extracellular enzyme activity were illustrated by RDA (Figure 4A). The first two axes of the RDA, explained 83.67 and 11.19% of the variation in the relationship between soil extracellular enzyme activities and SOM, TOC, TP, and AP in soil. The explained rates are 63.62 and 12.74% of the variation in the relationship between soil extracellular enzyme activities and more soil biochemicals, pH, TN, SOM, TOC, TP, AP, SK, DON, NO${}_{3}^{-}$, and NH${}_{4}^{+}$ (Supplementary Figure 3). With respective to soil matters, the confidence ellipses of organic fertilizer addition treatments separated from that of no fertilizer, where pig manure was significantly separated from other treatments.

After 12-year tobacco-maize rotation, the contents of SOM, TOC, TN, TP, AVP, SK, and CEC were the highest when PD were applied (Table 2). This could be associated with that long-term manure input can promote the accumulation of SOM by additional carbon inputs (Sehrish et al., 2021). However, organic fertilizer additions decreased the TK, DON, and EC (Supplementary Table 4). The content and accumulation of both ${\text{NH}}_{4}^{+}$ and NO${}_{3}^{-}$ tend to increase with applying organic fertilizer or nitrogen fertilizer (Li et al., 2020). In contrast, the NO${}_{3}^{-}$ was highest in no fertilization, probably due to nitrogen transfer to microorganisms through assimilation enhanced by fertilizers.

### Soil microorganisms and soil extracellular enzyme activity

The analysis of microbial phospholipid fatty acids (PLFAs) under different fertilizer treatments showed significant changes in soil microbial biomass (Supplementary Figures 4A–C and Supplementary Table 6) after 12-year tobacco-maize rotation. After applying inorganic fertilizer, the phospholipid fatty acid content of soil organisms and Gram-negative bacteria decreased (Supplementary Figures 4A,B). PD additions lead to the highest contents of the PLFAs for all microorganisms and Gram-negative bacteria. No significant differences in PLFA levels among multiple organic fertilizers additions are detected.

After 12-year tobacco-maize rotation, the PLFAs for actinomycetes are maximum as a result of PD addition (Supplementary Figure 4C). The differences between PLFAs for unspecific bacteria, Gram-positive bacteria, fungi, undefined organisms, and protozoan in different treatments are not significant (not shown). Inorganic fertilizer reduced the PLFAs contents of involved soil microorganisms. Therefore, animal-based fertilizers, PD addition had the most pronounced effect in PLFAs of soil microorganisms.

As shown in Figure 4B, the first two axes of the RDA, RDA1, and RDA2, explained 75.79 and 23.96% of the relationship between soil extracellular enzyme activities and the contents of PLFAs for the total organism, total Gram-negative bacteria, and total Actinomycetes. The content of PLFAs in OC addition differed significantly from other fertilizers and no fertilizer, illustrating by the confidence ellipse of OC addition separated from others.

The structure and function of the soil microbial population is influenced by cultivation and fertilization. The higher amount of microorganisms in PD addition, indicates the animal organic fertilizer PD more beneficial to the microbial function in the soil. GRSP, as a glycoprotein produced by fungal, contributes to soil organic carbon and nitrogen and the stabilization of soil particles (Liu et al., 2020). Our results found that the highest content of T-GRSP was in PD addition. Meanwhile, PD and S fertilizers induce more EE-GRSP than inorganic fertilizer, which agrees with the results that the organic fertilizer induces more GRSP in soil (Xie et al., 2015). Briefly, organic fertilizers promoted the growth of fungi and the production of GRSP in soil, this was exemplified by the arbuscular mycorrhizal fungi (Lovelock et al., 2004).

## Conclusion

Our findings demonstrated that, after 12-year tobacco-maize rotation, the long-term organic fertilizer addition could dramatically alter the nutrient levels in tobacco leaves indicating improved quality, *via* changing the activity of extracellular enzymes and soil nutrient levels. The different organic sources, thus the plant-based (oil cake and straw) and animal-based (pig dung) organic fertilizers differentially affect the activities of various soil enzymes. The relationships of extracellular enzyme activities with tobacco yield and quality, soil nutrient levels, and microorganisms, indicate that animal-based organic fertilizer, thus pig dung, should be used in combining with chemical fertilizers to improve the quality of tobacco and soil nutrients. Our study clarified the mechanisms behind soil biological changes in response to organic fertilizer additions in a tobacco-maize rotation system, which provided sufficient carbon and nitrogen sources for the activity of soil microorganisms activity. Farmers will be able to attain crop productivity and quality from this study.

## Data availability statement

The raw data supporting the conclusions of this article will be made available by the authors, without undue reservation.

## Author contributions

XD and W-FC designed the study. YJ performed the experiments and sample collections. RZ performed the data analysis. RZ and CZ wrote the manuscript. YJ, RZ, JS, and W-FC contributed to discussing the results and to finalizing manuscript. All authors contributed to the article and approved the submitted version.

## References

[B1] Bell C. W. Fricks B. E. Rocca J. D. Steinweg J. M. McMahon S. K. Wallenstein M. D. (2013). High-throughput fluorometric measurement of potential soil extracellular enzyme activities. *J. Vis. Exp.* 81:350961. 10.3791/50961 24299913 PMC3991303

[B2] Berzsenyi Z. Győrffy B. Lap D. (2000). Effect of crop rotation and fertilization on maize and wheat yields and yields stability in a long-term experiment. *Eur. J. Agron.* 13 225–244. 10.1016/S1161-0301(00)00076-9

[B3] Bossio D. A. Scow K. M. (1998). Impacts of carbon and flooding on soil microbial communities: Phospholipid fatty acid profiles and substrate utilization patterns. *Microb. Ecol.* 35 265–278. 10.1007/s002489900082 9569284

[B4] Bremner J. M. Mulvaney C. S. (1982). “Nitrogen-Total. In: Methods of soil analysis. Part 2. Chemical and microbiological properties,” in *American Society of Agronomy, Soil Science Society of America*, eds Page A. L. Miller R. H. Keeney D. R. (Wisconsin: Madison), 595–624.

[B5] Chen Y. Ren K. Su J. He X. Zhao G. Hu B. (2020). Rotation and organic fertilizers stabilize soil water-stable aggregates and their associated carbon and nitrogen in flue-cured tobacco production. *J. Soil Sci. Plant Nutr.* 20 192–205. 10.1007/s42729-019-00118-8

[B6] Chivenge P. Bernard V. Johan S. (2011). Dose the combined application of organic and mineral nutrient sources influence maize productivity? A meta-analysis. *Plant Soil* 342 1–30. 10.1007/s11104-010-0626-5

[B7] Cui Z. Chen C. Zhang L. Zheng P. Jin B. Hu Q. (2016). Effects of different phosphorus fertilizer applications on content of nitrogen, phosphorus and potassium, photosynthetic characteristics and yield of flue-cured tobacco. *J. Henan Agric. Univ.* 50 171–175. 10.16445/j.cnki.1000-2340.2016.02.005

[B8] Drope J. Schluger N. Cahn Z. Hamill S. Islami S. Liber A. (2018). *The Tobacco Atlas (sixth ed.).* Atlanta: American Cancer Society.

[B9] Farooq M. Hussain T. Wakeel A. Cheema Z. A. (2014). Differential response of maize and mungbean to tobacco allelopathy. *Exp. Agric.* 50 611–624. 10.1017/S0014479714000106

[B10] Frostegård Å Tunlid A. Bååth E. (1991). Microbial biomass measured as total lipid phosphate in soils of different organic content. *J. Microbiol. Methods* 14 151–163. 10.1016/0167-7012(91)90018-L

[B11] Galloway J. N. Townsend A. R. Erisman J. W. Bekunda M. Cai Z. Freney J. R. (2008). Transformation of the nitrogen cycle: Recent trends, questions, and potential solutions. *Science* 320 889–892. 10.1126/science.1136674 18487183

[B12] Guo D. Hu H. Liu X. Hou X. Shu J. Yao Z. (2014). Relationship between balance of chemical components and sensor quality of flue-cured tobacco. *J. Anhui Agric. Univ.* 41 333–337. 10.13610/j.cnki.1672-352x.20140225.0016

[B13] Hortal S. Bastida F. Armas C. Lozano Y. M. Moreno J. L. García C. (2013). Soil microbial community under a nurse-plant species changes in composition, biomass and activity as the nurse grows. *Soil Biol. Biochem.* 64 139–146. 10.1016/j.soilbio.2013.04.018

[B14] Jian S. Li J. Chen J. Wang G. Mayes M. A. Dzantor K. E. (2016). Soil extracellular enzyme activities, soil carbon and nitrogen storage under nitrogen fertilization: A meta-analysis. *Soil Biol. Biochem.* 101 32–43. 10.1016/j.soilbio.2016.07.003

[B15] Jiang Y. Zhang J. Manuel D. B. de Beeck M. O. Shahbaz M. Chen Y. (2022). Rotation cropping and organic fertilizer jointly promote soil health and crop production. *J. Enivr. Manag.* 315:115190. 10.1016/j.jenvman.2022.115190 35526398

[B16] Kalembasa S. J. Jenkinson D. S. (1973). A comparative study of titrimetric and gravimetric methods for the determination of organic carbon in soil. *J. Sci. Food Agric.* 24 1085–1090. 10.1002/jsfa.2740240910

[B17] Li J. Shao X. Huang D. Shang J. Liu K. Zhang Q. (2020). The addition of organic carbon and nitrogen accelerates the restoration of soil system of degraded alpine grassland in Qinghai-Tibet Plateau. *Ecol. Eng.* 158:106084. 10.1016/j.ecoleng.2020.106084

[B18] Li X. Liu F. Wang H. He F. Yang R. Zhao M. (2019). Gas chromatography-mass spectrometry method for simultaneous detection of nine alkaloids in tobacco and tobacco products by QuEChERS sample preparation. *Anal. Sci.* 35 849–854. 10.2116/analsci.19P063 30930354

[B19] Liu H. Du X. Li Y. Han X. Li B. Zhang X. (2022). Organic substitutions improve soil quality and maize yield through increasing soil microbial diversity. *J. Clean. Prod.* 347:131323. 10.1016/j.jclepro.2022.131323

[B20] Liu H. Wang X. Liang C. Ai Z. Wu Y. Xu H. (2020). Glomalin-related soil protein affects soil aggregation and recovery of soil nutrient following natural revegetation on the Loess Plateau. *Geoderma* 357:113921. 10.1016/j.geoderma.2019.113921

[B21] Liu T. Guo L. Cao C. Tan W. Li C. (2021). Long-term rice-oilseed rape rotation increases soil organic carbon by improving functional groups of soil organic matter. *Agric. Ecosyst. Environ.* 319:107584. 10.1016/j.agee.2021.107548

[B22] Lourenço K. S. Suleiman A. K. A. Pijl A. Cantarella H. Kuramae E. E. (2020). Dynamics and resilience of soil mycobiome under multiple organic and inorganic pulse disturbances. *Sci. Total Environ.* 733:139173. 10.1016/j.scitotenv.2020.139173 32454291

[B23] Lovelock C. E. Wright S. F. Clark D. A. Ruess R. W. (2004). Soil stocks of glomalin produced by arbuscular mycorrhizal fungi across a tropical rain forest landscape. *J. Ecol.* 92 278–287. 10.1111/j.0022-0477.2004.00855.x

[B24] Lupwayi N. Z. Zhang Y. Hao X. Thomas B. W. Eastman A. H. Schwinghamer T. D. (2019). Linking soil microbial biomass and enzyme activities to long-term manure applications and their nonlinear legacy. *Pedobiologi* 74 34–42. 10.1016/j.pedobi.2019.04.001

[B25] Mauclaire L. Pelz O. Thullner M. Abraham W. R. Zeyer J. (2003). Assimilation of toluene carbon along a bacteria–protist food chain determined by 13C-enrichment of biomarker fatty acids. *J. Microbiol. Methods* 5 635–649. 10.1016/S0167-7012(03)00205-7 14607407

[B26] Palm C. A. Gachengo C. N. Delve R. J. Cadisch G. Giller K. E. (2001). Organic inputs for soil fertility management in tropical agroecosystems: Application of an organic resource database. *Agric. Ecosyst. Environ.* 83 27–42. 10.1016/S0167-8809(00)00267-X

[B27] Saleque M. A. Abedin M. J. Bhuiyan N. I. Zaman S. K. Panaullah G. M. (2004). Long-term effects of inorganic organic fertilizer sources on yield and nutrient accumulation of lowland rice. *Field Crops Res.* 86 53–65. 10.1016/S0378-4290(03)00119-9

[B28] Schmitt A. Glaser B. (2011). Organic matter dynamics in a temperate forest soil following enhanced drying. *Soil Biol. Biochem.* 43 478–489. 10.1016/j.soilbio.2010.09.037

[B29] Serra S. Morgante L. (1980). Method of determination of proteins with Coomassie brilliant blue G 250. I. General characteristics and comparative analysis with the biuret method and Lowry’s method. *Boll. Soc. Ital. Biol. Sper.* 56 160–165. 6159906

[B30] Sehrish A. Li D. Huang J. Waqas A. Muhammad A. Muhammad Q. (2021). Soil microbial biomass and extracellular enzymes regulate nitrogen mineralization in a wheat-maize cropping system after three decades of fertilization in a Chinese Ferrosol. *J. Soils Sediments* 21, 281–294. 10.1007/s11368-020-02770-5

[B31] Sinsabaugh R. L. Hill B. H. Follstad Shah J. J. (2009). Ecoenzymatic stoichiometry of microbial organic nutrient acquisition in soil and sediment. *Nature* 462 795–798. 10.1038/nature08632 20010687

[B32] Soil Survey Staff (2014). *Keys to soil taxonomy*, 12th edn. Washington, DC: USDA-Natural Resources Conservation Service.

[B33] Stedman R. L. (1968). The chemical composition of tobacco and tobacco smoke. *Chem. Rev.* 68 153–207. 10.1021/cr60252a002 4868017

[B34] Su J. Q. Ding L. J. Xue K. Yao H. Y. Quensen J. Bai S. J. (2015). Long-term balanced fertilization increases the soil microbial functional diversity in a phosphorus-limited paddy soil. *Mol. Ecol.* 24 136–150. 10.1111/mec.13010 25410123

[B35] Sun J. He J. Wu F. Tu S. Yan T. Si H. (2011). Comparative analysis on chemical components and sensory quality of aging flue-cured tobacco from four main tobacco areas of China. *Agric. Sci. China* 10 1222–1231. 10.1016/S1671-2927(11)60113-2

[B36] Tian K. Zhao Y. Xu X. Hai N. Huang B. Deng W. (2015). Effects of long-term fertilization and residue management on soil organic carbon changes in paddy soils of China: A meta-analysis. *Agric. Ecosyst. Environ.* 204 40–50. 10.1016/j.agee.2015.02.008

[B37] Turšić I. Butorac A. Bašić F. Čavlek M. Mesić M. Djaković Z. (2005). Effect of fifteen years of tobacco production in monoculture and in crop rotation on the yield and quality of flue-cured tobacco under agroecological conditions of Croatia: 15 years of long term investigation. *Die Bodenkultur* 56 169–172.

[B38] Vance E. D. Brookes P. C. Jenkinson D. S. (1987). An extraction method for measuring soil microbial biomass C. *Soil Biol. Biochem.* 19 703–707. 10.1016/0038-0717(87)90052-6

[B39] Vepsäläinen M. Kukkonen S. Vestberg M. Sirviö H. Niemi R. M. (2001). Application of soil enzyme activity test kit in a field experiment. *Soil Biol. Biolchem.* 33 1665–1672. 10.1016/S0038-0717(01)00087-6

[B40] Waldrop M. P. Zak D. R. Sinsabaugh R. L. (2004). Microbial community response to nitrogen deposition in northern forest ecosystems. *Soil Biol. Biochem.* 36 1443–1451. 10.1016/j.soilbio.2004.04.023

[B41] Xie H. Li J. Zhang B. Wang L. Wang J. He H. (2015). Long-term manure amendments reduced soil aggregate stability via redistribution of the glomalin-related soil protein in macroaggregates. *Sci. Rep.* 5:14687. 10.1038/srep14687 26423355 PMC4589770

[B42] Yang W. Wang L. Xu Z. Li Q. Jiao J. (2011). Analysis of relationships between potassium content, chlorine content, ratio of potassium to chlorine in flue-cured tobacco leaves and smoking gas indices. *Acta Agric. Jiangxi* 23 109–112. 10.19386/j.cnki.jxnyxb.2011.12.033

[B43] Zhang J. Sun X. (2021). Recent advances in polyphenol oxidase-mediated plant stress responses. *Phytochemistry* 181:112588. 10.1016/j.phytochem.2020.112588 33232863

[B44] Zhang Z. Zhang Z. Mahamood M. D. Zhang S. Huang S. Liang W. (2016). Effect of long-term combined application of organic and inorganic fertilizers on soil nematode communities within aggregates. *Sci. Rep.* 6:31118. 10.1038/srep31118 27502433 PMC4977470

[B45] Zheng L. Chen H. Wang Y. Mao Q. Zheng M. Su Y. (2020). Responses of soil microbial resource limitation to multiple fertilization strategies. *Soil Tillage Res.* 196:104474. 10.1016/j.still.2019.104474

[B46] Zou C. Pearce R. C. Grove J. H. Coyne M. S. (2015). Conservation practices in tobacco production increase large aggregates an associated carbon and nitrogen. *Soil Sci. Soc. Am. J.* 79:1760. a 10.2136/sssaj2015.06.0235

