# Supplementary material for: Long-term organic fertilizer additions elevate soil extracellular enzyme activities and tobacco quality in a tobacco-maize rotation
Source: Front Plant Sci. 2022 Sep 9;13:973639. doi: 10.3389/fpls.2022.973639 (PMC9501973; doi:10.3389/fpls.2022.973639)
Supplement: Supplementary file 1 [file Data_Sheet_1.pdf]

Supplementary Material

Long-term organic additions elevate soil extracellular enzyme activities and tobacco quality in a tobacco-maize rotation

Yonglei Jiang, Ruqiang Zhang, Cuiping Zhang, Jiaen Su, Wen-Feng Cong, Xiaopeng Deng

1 Supplementary Figures

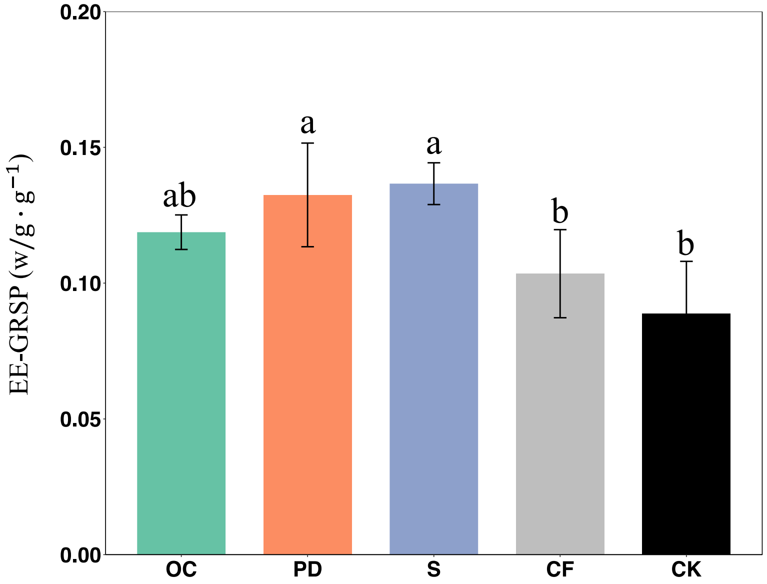

Supplementary Figure 1. Effects of different fertilization management on the activities of T-GRSP after a 12-year tobacco-maize rotation. T-GRSP, total glomalin-related soil protein. OC, oil cake and inorganic nitrogen; PD, pig dung and inorganic nitrogen; S, straw and inorganic nitrogen; CF, inorganic nitrogen; CK, no fertilization. Values are means ± SE, n=3. Different lowercase letters indicate significant differences among fertilizer treatments ($P < 0.05$).

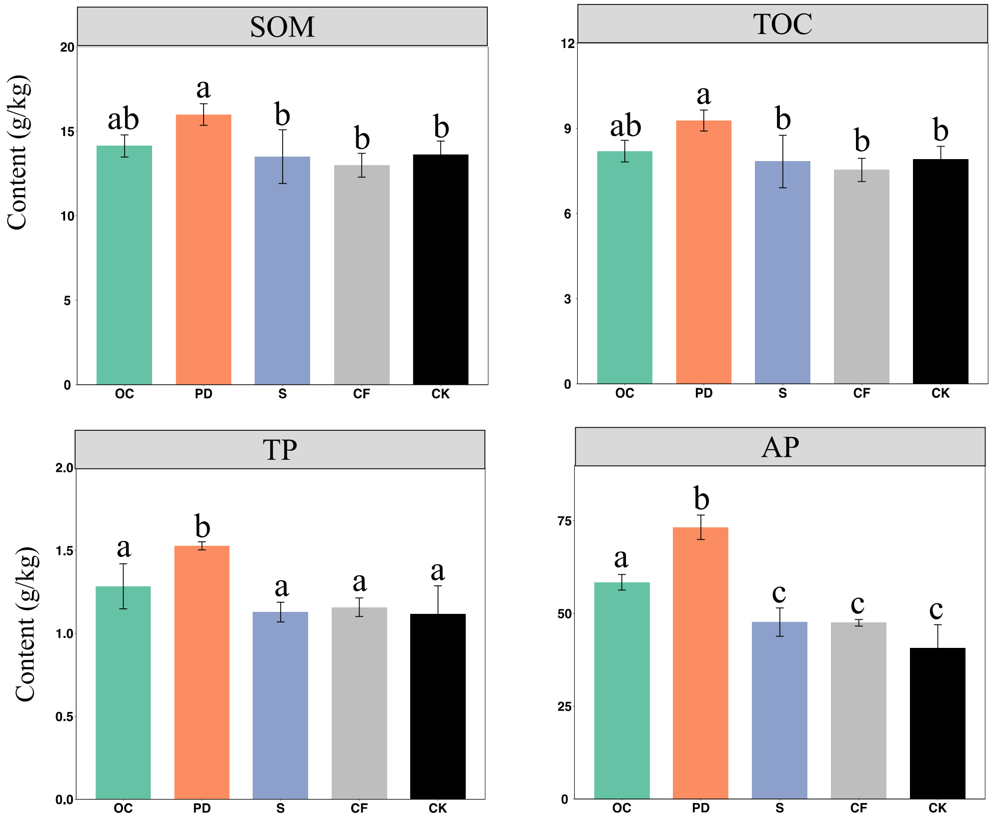

Supplementary Figure 2. The contents of soil organic matter (SOM), total organic carbon (TOC), total phosphorus (TP), available phosphorus (AVP) in soil under fertilizations oil cake (OC), pig manure (PD), straw (S), chemical (CF) and no fertilization (CK) after a 12-year tobacco-maize rotation. See Supplementary Materials for more contents of chemicals in soil. Data are means ± SE. Significant differences were determined by ANOVA, Fisher's least significant difference (LSD) test (*P*<0.05) and indicated by different letters.

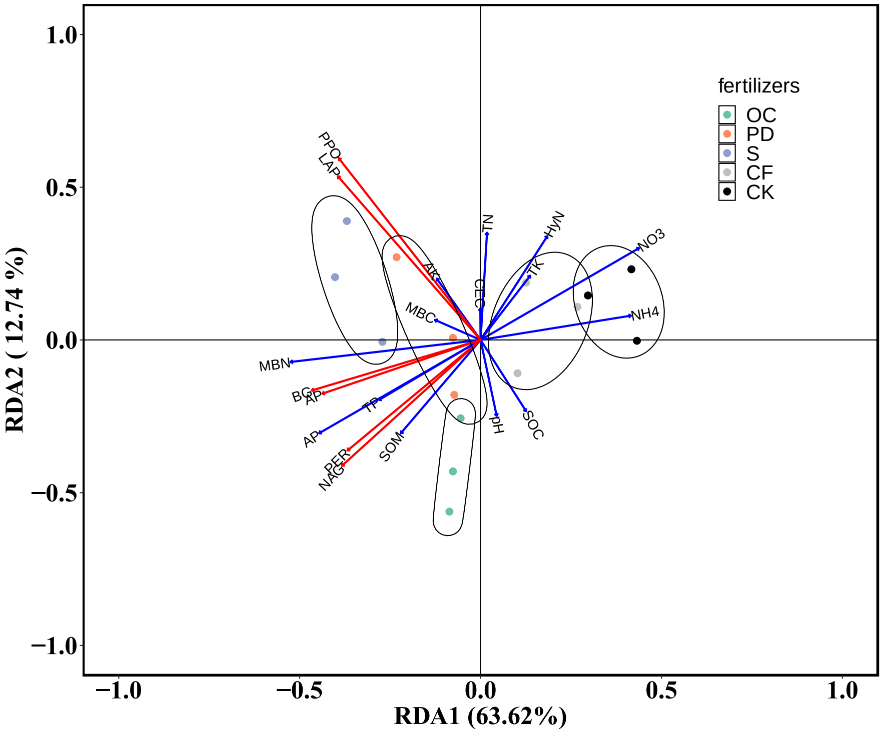

Supplementary Figure 3. Redundancy analysis (RDA) of extracellular enzyme activity on soil properties after a 12-year tobacco-maize rotation. pH, soil pH; TN, total nitrogen; SOM, soil organic matter; TOC, total organic carbon; TP, total phosphorus; AVP, available phosphorus; SK, available potassium; DON, dissolved organic nitrogen; NO3, nitrate nitrogen; NH4, ammonium nitrogen; BG, β-1, 4-glucosidase; NAG, β-1,4-N-acetyl-glucosaminidase; LAP, L-leucine aminopeptidase; AP, acid phosphatase; PER, peroxidase; PPO, polyphenol oxidase. OC, oil cake and inorganic nitrogen; PD, pig dung and inorganic nitrogen; S, straw and inorganic nitrogen; CF, inorganic nitrogen; CK, no fertilization.

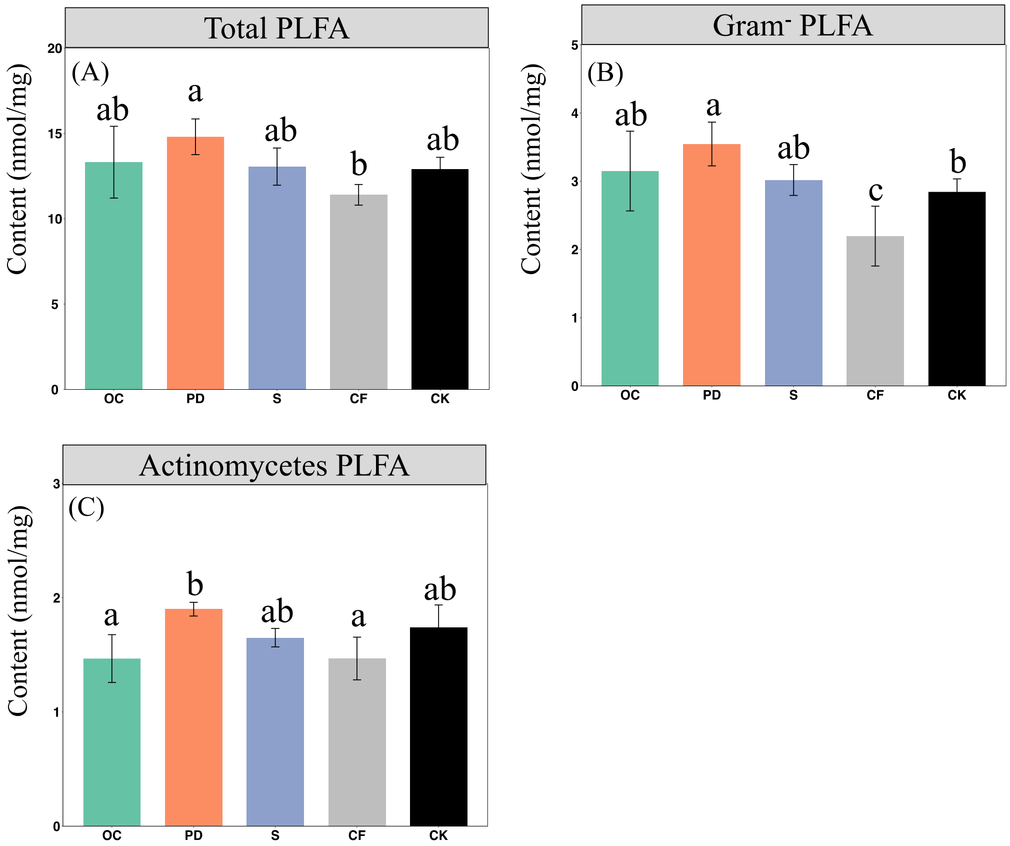

Supplementary Figure 4. The contents of PLFAs for total organisms (A), total Gram-negative bacteria (B), total *Actinomycetes* (C) in soil under fertilizations oil cake (OC), pig manure (PD), straw (S), chemical (CF) and no fertilization (CK) after a 12-year tobacco-maize rotation. OC, oil cake and inorganic nitrogen; PD, pig dung and inorganic nitrogen; S, straw and inorganic nitrogen; CF, inorganic nitrogen; CK, no fertilization. See Supplementary Materials for more contents of organisms the in soil. Data are means ± SE. Significant differences were determined by ANOVA, Fisher's least significant difference (LSD) test (P<0.05) and indicated by different letters.

2 Supplementary Tables

Supplementary Table 1

Basic physicochemical properties of organic fertilizers.

Fertilizer	total C (%)	total N (%)	total P (%)	total K (%)	organic matter (%)	pH	water content (%)	C/N ratio
oil cake	50.52	5.13	2.1	1.28	87.1	5.86	12.6	9.85
pig dung	23.05	1.55	5.12	2.82	67.93	7.52	21.7	14.87
straw	43.69	0.88	0.11	1.44	75.32	8.47	8.2	49.65

Supplementary Table 2

List of extracellular enzymes in soil, corresponding substrates and their functions.

Enzyme	Substrate	Abbr.	Function
β-1,4-glucosidase	4-MUB-β-D-glucoside	BG	Hydrolysis of cellobioside
β-1,4-N-acetylglucosaminidase	4-MUB-N-acetyl-β-D-glucosaminide	NAG	Hydrolysis of chin
L-leucine aminopeptidase	L-leucine-7-amino-4-methylcoumarin	LAP	Hydrolysis of protein
Acid phosphatase	4-MUB-phosphate	AP	Hydrolysis of organic P
Polyphenol oxidase	1,2-dihydroxybenzene	PPO	Lignin-degrading enzymes, oxidizes phenolic compounds
Peroxidase	L-DOPA	PER	Oxidation lignin

Supplementary Table 3

P values for analysis of variance (ANOVA) for the main and interactive effects of fertilization and parts on element levels in tobacco.

Element levels	Fertilization	Leaf Positions	Fertilization × Parts
Total sugar (%)	0.031*	0.069	0.2145
Reductive sugar (%)	0.046	0.612	0.430
Total alkaline (%)	<0.010**	<0.010**	0.797
Total nitrogen (N, %)	0.048*	<0.010**	0.715
Potassium (K_2O, %)	<0.010**	0.685	0.900
Phosphorus (P, g/100g)	0.018*	0.521	0.999

**$p < 0.01$; *$p < 0.05$.

Supplementary Table 4

Effects of different fertilizations on soil organic matter, carbon, nitrogen, phosphorus, potassium after a 12-year tobacco-maize rotation.

Fertilization management	SOM (g/kg^{-1})	TOC (g/kg^{-1})	TN (g/kg^{-1})	TP (g/kg^{-1})	TK (g/kg^{-1})	NO_3^- (g/kg^{-1})	NH_4^+ (g/kg^{-1})	DON (g/kg^{-1})	AVP (g/kg^{-1})	SK (mg/kg^{-1})	CEC (cmol/kg)	EC (%)
OC	14.1±0.4B	8.2±0.2B	0.9±0.06B	1.3±0.08B	7.5±0.3A	70.4±13.2A	2.1±0.5AB	85.5±4.7A	58.4±1.2B	151.4±17.6A	6.4±0.6A	145.5±18.4B
PD	16.0±0.4A	9.3±0.2A	1.2±0.1A	1.5±0.02A	7.2±0.5A	90.4±31.1A	2.9±1.2AB	114.9±11.5A	73.2±1.9A	203.8±13.1A	6.9±0.5A	318.2±66.3AB
S	13.5±0.9B	7.8±0.5B	1.2±0.04A	1.1±0.03B	7.4±0.5A	47.3±6.4A	0.3±0.08B	88.8±4.9A	47.7±2.2C	166.3±28.8A	6.2±0.3A	225.5±38.8AB
CF	13.0±0.4B	7.5±0.2B	1.0±0.05AB	1.2±0.03B	7.7±0.4A	158.1±68.6A	4.3±1.1A	116.9±11.0A	47.5±0.5C	174.6±16.0A	6.4±0.1A	455.7±88.2A
CK	13.6±0.5B	7.9±0.3B	1.1±0.1AB	1.1±0.1B	7.6±0.4A	174.8±113.3A	2.9±1.2AB	100.1±12.2A	40.6±3.7C	152.6±48.2A	6.8±0.2A	339.4±146.9AB

Value are means ± SE, n=3. Different capital letters indicate significant among fertilizer treatments (P < 0.05). OC, oil cake and inorganic nitrogen; PD, pig dung and inorganic nitrogen; S, straw and inorganic nitrogen; CF, inorganic nitrogen; CK, no fertilization.

Supplementary Table 5

Effects of different fertilizations on soil microbial biomass of Carbon, Nitrogen and Phosphorus after a 12-year tobacco-maize rotation.

Fertilization management	MBC ($mg\ kg^{-1}$)	MBN ($mg\ kg^{-1}$)	MBP ($mg\ kg^{-1}$)	MBC:N	MBC:P	MBN:P
OC	98.7±6.2A	42.4±3.2A	2.5±0.6A	2.4±0.2A	43.7±7.2AB	19.2±4.6AB
PD	102.0±14.0A	36.1±5.4A	3.0±0.2A	2.9±0.4A	33.8±3.3AB	12.1±2.2B
S	89.0±16.9A	41.8±3.2A	1.4±0.1A	2.2±0.6A	64.2±6.5A	31.5±4.8A
CF	96.7±16.0A	36.3±5.7A	3.5±1.6A	2.7±0.6A	44.7±19.6AB	16.6±8.4Ab
CK	82.8±11.7A	26.3±8.3A	4.2±1.4A	3.5±0.6A	25.5±8.7B	7.8±2.6B

Value are means ± SE, n=3. Different capital letters indicate significant among fertilizer treatments ($P < 0.05$). MBC, soil microbial biomass carbon; MBN soil microbial biomass nitrogen; MBP, soil microbial biomass phosphorus. OC, oil cake and inorganic nitrogen; PD, pig dung and inorganic nitrogen; S, straw and inorganic nitrogen; CF, inorganic nitrogen; CK, no fertilization.

Supplementary Table 6

Microbial community composition PLFAs (nmol g^{-1}) under different fertilization treatments. Values (means ± SE, n=3) followed by different letters indicate significant difference among soil stages detected by Tukey's HSD tests (*P* < 0.05). ND, Not detected.

PLFA maker		OC	PD	S	CF	CK
Unspecific bacterical	12:00	0.015±0.002ab	0.018±0.001ab	0.015±0.004ab	0.020±0.006a	0.013±0.003b
	13:00	0.011±0.006a	0.013±0.001a	0.017±0.005a	0.019±0.002a	0.012±0.002a
	14:00	0.114±0.014a	0.125±0.001a	0.110±0.010a	0.110±0.003a	0.104±0.001a
	15:1 iso w6c	0.122±0.012a	0.111±0.006a	0.113±0.001a	0.110±0.011a	0.101±0.007a
	15:1 anteiso w9c	0.048±0.008a	0.037±0.005ab	0.035±0.0002ab	0.033±0.004ab	0.030±0.004b
	15:00	0.118±0.0013a	0.107±0.006a	0.096±0.009a	0.099±0.010a	0.097±0.006a
	16:00	1.371±0.158a	1.375±0.045a	1.153±0.105ab	1.039±0.064b	1.201±0.054ab
	17:1 anteiso w7c	0.041±0.004a	0.033±0.006a	0.036±0.0004a	0.035±0.003a	0.030±0.004a
	17:00	0.109±0.009a	0.109±0.005a	0.100±0.009a	0.088±0.006a	0.090±0.007a
	18:00	0.336±0.032ab	0.364±0.021a	0.295±0.021bc	0.271±0.003c	0.310±0.004abc
	17:0 iso 3OH	0.053±0.010b	0.065±0.005a	0.056±0.002b	0.047±0.006b	0.060±0.004ab
Gram+	13:0 iso	0.017±0.004a	0.020±0.002a	0.022±0.003a	0.018±0.002a	0.019±0.005a
	13:0 anteiso	0.018±0.002a	0.019±0.002a	0.020±0.002a	0.020±0.003a	0.018±0.002a
	14:0 iso	0.068±0.012a	0.085±0.003a	0.069±0.008a	0.065±0.005a	0.074±0.003a
	14:0 anteiso	0.027±0.005a	0.026±0.001a	0.028±0.003a	0.032±0.004a	0.028±0.002a
	15:0 iso	0.846±0.084a	1.001±0.054a	0.917±0.55a	0.833±0.058a	0.925±0.023a
	15:0 anteiso	0.546±0.063a	0.573±0.035a	0.5020.034a	0.465±0.021a	0.473±0.025a
	16:0 iso	0.583±0.052ab	0.617±0.033a	0.545±0.034ab	0.487±0.019b	0.503±0.032b

	16:0 anteiso	0.106±0.003a	0.089±0.010ab	0.089±0.004ab	0.089±0.004ab	0.082±0.009b
	17:1 iso w9c	0.599±0.011a	0.594±0.059a	0.575±0.003a	0.562±0.035a	0.556±0.039a
	17:0 iso	0.291±0.034a	0.299±0.013a	0.266±0.016a	0.247±0.009a	0.264±0.012a
	17:0 anteiso	0.353±0.030ab	0.371±0.021a	0.332±0.018ab	0.296±0.016b	0.312±0.024ab
	18:0 iso	0.086±0.010a	0.094±0.006a	0.085±0.006a	0.129±0.058a	0.087±0.004a
	19:0 iso	0.047±0.012a	0.045±0.008a	0.032±0.008a	0.033±0.005a	0.038±0.005a
Gram-	12:0 2OH	0.014±0.008a	0.016±0.003a	0.013±0.007a	0.011±0.006a	0.006±0.006a
	14:1 w5c	0.018±0.004a	0.019±0.0002a	0.020±0.002a	0.022±0.002a	0.019±0.002a
	15:1 w6c	0.034±0.004a	0.033±0.007a	0.034±0.007a	0.033±0.003a	0.031±0.006a
	15:1 w5c	0.016±0.001a	0.012±0.002ab	0.016±0.002a	0.010±0.005ab	0.004±0.004b
	16:1 w9c	0.087±0.008c	0.121±0.001a	0.112±0.010ab	0.089±0.010bc	0.110±0.007abc
	16:1 w7c	0.742±0.064ab	0.895±0.063a	0.766±0.015ab	0.614±0.030b	0.686±0.053b
	16:1 w7c DMA	0.068±0.002a	0.052±0.009ab	0.059±0.002ab	0.057±0.004ab	0.032±0.018b
	17:1 w8c	0.137±0.005ab	0.1710.017a	0.157±0.006ab	0.124±0.003b	0.152±0.023ab
	17:0 cyclo w7c	0.384±0.044a	0.3667±0.014ab	0.305±0.022abc	0.286±0.035bc	0.271±0.013c
	16:0 2OH	0.016±0.006a	0.009±0.005a	0.011±0.001a	0.011±0.006a	ND
	18:1 w7c	0.877±0.115a	1.073±0.068a	0.86±0.039a	0.42±0.212b	0.868±0.028a
	18:1 w5c	0.243±0.034ab	0.297±0.019a	0.248±0.018ab	0.135±0.069b	0.256±0.015a
	19:0 cyclo w7c	0.511±0.074a	0.482±0.033ab	0.415±0.026ab	0.383±0.021b	0.408±0.014ab
Undefined microbia	15:4 w3c	0.007±0.007b	0.024±0.002a	0.025±0.001a	0.015±0.008ab	0.023±0.002a
	15:0 DMA	0.185±0.025a	0.194±0.012a	0.178±0.010a	0.164±0.006a	0.174±0.018a
	16:3 w6c	0.027±0.014ab	0.009±0.009b	NONE	0.022±0.011ab	0.041±0.006a

	17:1 w7c 10-methyl	0.104±0.052a	0.177±0.010a	0.160±0.005a	0.147±0.010a	0.167±0.012a
	18:1 w7c 10-methyl	0.115±0.024a	0.113±0.006a	0.089±0.006ab	0.073±0.003b	0.083±0.002ab
	18:1 w7c DMA	0.028±0.008a	0.025±0.004ab	0.009±0.005bc	0.016±0.004ab	0.008±0.008b
	19:3 w6c	0.044±0.015a	0.026±0.013a	0.040±0.008a	0.022±0.004a	0.035±0.001a
	19:1 w8c	0.036±0.003a	0.028±0.014a	0.036±0.001a	0.030±0.001a	0.035±0.004a
	19:1 w7c 10-methyl	0.008±0.006a	0.008±0.008a	0.007±0.005a	0.005±0.005a	0.006±0.006a
	20:5 w3c	0.017±0.004b	0.045±0.007a	0.022±0.006b	0.016±0.003b	0.021±0.003b
	20:3 w6c	0.028±0.002a	0.030±0.004a	0.030±0.007a	0.020±0.003a	0.020±0.003a
Actinomycetes	16:0 10-methyl	0.818±0.063b	1.103±0.045a	0.980±0.049ab	0.844±0.089b	1.000±0.062ab
	17:0 10-methyl	0.249±0.022a	0.270±0.029a	0.240±0.005a	0.220±0.013a	0.232±0.021a
	18:0 10-methyl	0.401±0.039c	0.528±0.019a	0.430±0.006bc	0.404±0.029c	0.506±0.035ab
Fungi	16:1 w5c(AMF)	0.300±0.032b	0.412±0.004a	0.379±0.033a	0.297±0.015b	0.360±0.027ab
	18:3 w6c	0.115±0.003a	0.096±0.014a	0.093±0.006	0.131±0.038	0.086±0.008a
	18:2 w6c	0.308±0.044a	0.301±0.037	0.250±0.018	0.321±0.120a	0.398±0.105a
	18:1 w9c	0.709±0.047b	0.897±0.043a	0.736±0.048b	0.662±0.047b	0.760±0.051ab
	18:1 w9c DMA	0.021±0.006a	0.020±0.004a	0.013±0.0002a	0.009±0.004a	0.011±0.006a
	20:1 w9c	0.069±0.002b	0.099±0.007a	0.088±0.005a	0.053±0.007b	0.067±0.003b
Protozoan	20:4 w6c	0.084±0.018a	0.105±0.007a	0.088±0.017a	0.072±0.009a	0.083±0.003a
	20:00	0.052±0.012a	0.073±0.004a	0.065±0.005a	0.057±0.007a	0.065±0.001a

OC, oil cake and inorganic nitrogen; PD, pig dung and inorganic nitrogen; S, straw and inorganic nitrogen; CF, inorganic nitrogen; CK, no fertilization.
